# Risk factors for cardiovascular and cerebrovascular diseases among ethnic Germans from the former Soviet Union: results of a nested case-control study

**DOI:** 10.1186/1471-2458-12-190

**Published:** 2012-03-13

**Authors:** Ema Kuhrs, Volker Winkler, Heiko Becher

**Affiliations:** 1 Institute of Public Health, University of Heidelberg, Im Neuenheimer Feld 324, Heidelberg 69120, Germany

**Keywords:** Nested case-control study, Risk factors, CVD-Mortality, Migrants, Aussiedler, Soviet Union

## Abstract

**Background:**

Diseases of the circulatory system (CVD) are the most common causes of death in developed countries. However, the prevalence of CVD varies between countries; for example, the mortality rate in Russia is about four times higher than in Western Europe. In a recent retrospective cohort study it was unexpectedly found that CVD mortality is lower among "Aussiedler" (ethnic Germans from the former Soviet Union) compared to the German population.

**Methods:**

This is a case-control study, nested into a recent cohort study of migrants from the former Soviet Union. Relatives of cases and controls themselves were interviewed by telephone using a standardized questionnaire. To estimate relative risks via the odds ratio (OR), a conditional logistic regression procedure was performed.

**Results:**

Commonly known risk factors for CVD were identified as relevant to Aussiedler. The best multivariate model for CVD includes five risk factors: consumption of alcohol, smoking, diabetes, cholesterol and consumption of sweets. For alcohol consumption and smoking, OR = 3.68 (95% CI, 1.58-8.58) and OR = 3.07 (95% CI, 1.42-6.62), respectively. For diabetes mellitus and high cholesterol values, OR = 3.29 (95% CI, 1.50-7.39) and OR = 2.32 (95% CI, 1.11-4.88), respectively. The almost complete abdication of sweets is associated with a protective effect, OR = 0.34 (95% CI, 0.18-0.64). The prevalence of risk factors is somewhat different to that of the autochthon German population and partly explains the differences in CVD mortality between both groups.

**Conclusions:**

The reported lower prevalences of known risk factors of CVD such as alcohol consumption, high cholesterol, diabetes and smoking (in women) could contribute to a lower risk of CVD.

## Background

Migration is an old phenomenon that continues to this day. Germany is a country of destination for migrants from many different parts of the world, and migrants constitute 9% percent of the total population of Germany [1]. The study of migrant health is an important challenge in health care. Migrants are often subject to different risks and can differ in their health awareness and behavior than the population of the host country, and consequently can have different morbidity and mortality [2].

**Figure 1:**
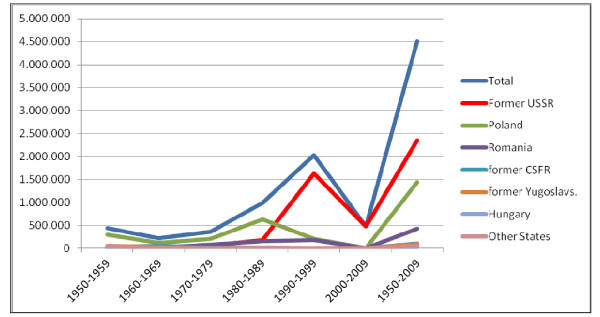
**The number of ethnic Germans for Countries of origin 1950-2009**. Excerpt from the Federal Administrative Office, Annual Statistics 2009, p. 5.

Between 1950 and 2009, approximately 4.5 million migrants came to Germany (Figure 1). Of these, more than two million came from the territory of the former Soviet Union [3]. They are ethnic Germans, so called "Aussiedler" or "Spätaussiedler" (resettlers).

To date, little reliable information about the health behavior of migrants from the countries of the former Soviet Union is available in epidemiological studies [4,5]. Studies on the migration itself and its impact on health are of great interest, as it is acknowledged that there is a connection between health and migration [6]. This can be due to communication problems and past experiences that change attitudes to health and disease [7-9]. An investigation based on cooperative health research in the Augsburg region (KORA study) [4] shows that migrants assess their health as poorer, that they participate inadequately in measures of health care and that they are more likely to be overweight and less physically active than Germans. After the collapse of the Soviet Union, the death rate among Russians increased rapidly [10-14]. In 2007, the total mortality of the population in Russia was 1465 per 100,000 inhabitants. Of these, more than 50% were caused by diseases of the circulatory system (ICD10 -I00-I99) [15]. Low socio-economic status was associated with increased cardiovascular mortality in many studies in different populations [16-18]. Because of the high cardiovascular mortality in the Russian population and the generally low socio-economic status of migrants, it has been suggested that cardiovascular mortality should be greater among Aussiedler than among the native Germans [19]. However, in a recent retrospective cohort study it was shown that the cardiovascular mortality among Aussiedler was not only lower in comparison to the mortality in Russia, but also lower in comparison to the mortality of the general population of Germany with a standardized mortality ratio, SMR_cvd_, of 0.79 [95%-CI 0.73-0.85] [11,4].

The aim of this study is to identify and to quantify cardiovascular and cerebrovascular diseases risk factors, and to compare the prevalence of these factors to that of the German population.

## Materials and methods

### Study design and study population

**Figure 2:**
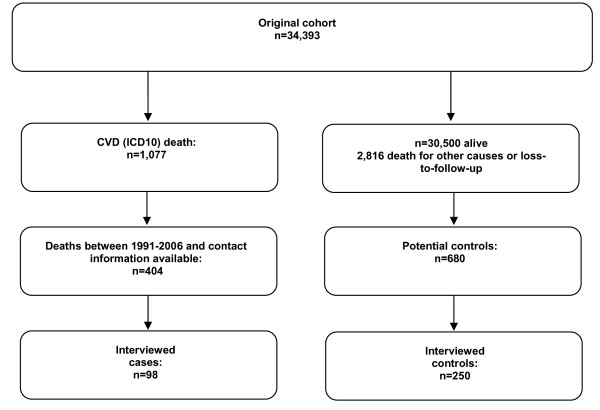
**Selection of cases and controls from the original cohort**.

This is a case-control study, nested into a recent cohort study of migrants from the former Soviet Union [4]. The original cohort consists of 34,393 out of 281,356 migrants with the age of at least 15 years at entry to North Rhine-Westphalia (NRW) in the period between 01.01.1990 and 31.12.2001 for whom an automated record linkage with the population registry of their first residence was possible (for the detailed description of the cohort see [5,20]). For the present nested case-control study, all deaths from cardiovascular diseases within the cohort were considered. Figure 2 describes the selection of cases and controls from the original cohort. Until the end of the follow-up on 31.12.2005, 1077 deaths from cardiovascular diseases (International Statistical Classification of Diseases and Related Health Problems ICD10 - I00-I99) were observed. The causes of death were coded according to ICD-9 and ICD-10 depending on year of death. For 404 cases, one or more relatives were available within the cohort. To ensure comparability between cases and controls, cases were matched using the propensity score [21-24] by year of birth, immigration year, and gender. A 1:4 matching was originally planned, however, an insufficient number of potential controls were available for several cases which fulfilled the matching criteria. A total of 680 controls from the original cohort were selected from all survivors of the cohort according to the matching criteria and approached via an invitation letter and telephone contact (if telephone number was available).

### Data collection

Relatives of cases and controls themselves were interviewed by telephone using a standardized questionnaire. To ensure comparability relatives of cases and controls were both asked first about their own risk factors, followed by questions of deceased relatives. The questionnaire was developed on the basis of the KORA study and tested on randomly selected individuals [25]. In total, the questionnaire consists of 45 closed questions from the following subject areas: Socio-demography and profession: sex, date of birth, country of origin, immigration year, school and university degrees, profession, retirement age; Health: subjective assessment of their physical constitution and issues with existing illnesses; Lifestyle factors: smoking habits, alcohol consumption, nutrition and sports activities; Anthropometric information and Family history of CVD and cancer.

For recruitment of participants, the following measures were taken: Bilingual questionnaire and a bilingual interviewer (German and Russian), contact at desired times, interviewing time not longer than 20 minutes, five euro compensation offer, reminder letter after two weeks, second reminder letter with advertising gift and newspaper interview with the project manager from the magazine "KOHTAKT-IIIAHC", issue No. 21 dated 22.05.2006, as well as another personal contact by telephone. Data collection took place between August 2009 and February 2010 for cases and between February 2010 and September 2010 for controls.

### Participant information and consent form

Before contact was established, the content of the survey and the data protection rules were explained to potential participants by sending a consent form and information material. By signing the consent form or by a telephone call, the participants declared willingness to participate and assured that they were informed sufficiently on the study and the further use of the data collected. Participation could be withdrawn at any time.

### Ethical aspects

The study was approved by the ethics committee of the medical faculty of Heidelberg University.

### Statistical analysis

After data processing, analysis was carried out using statistical analysis system version 9.2 (SAS 9.2). Data analysis was descriptive and analytical. To estimate relative risks via the odds ratio (OR), a conditional logistic regression procedure was performed [26]. First, univariate analysis was conducted to examine individual risk factors. For the multivariate logistic regression model, the following potential risk factors were considered: high body mass index (BMI > 27.0 kg/m^2^), high alcohol consumption (> 0.76 L per month [27]), smoking (ever vs. never smoking), sports (intensive sports activities vs. no activities), consumption of fruits and vegetables (daily or several times a week vs. less consumption), consumption of sweets, salt intake, burden of work (heavy physical work), education, medical history (diabetes mellitus, hypertension, elevated cholesterol levels, drug treatment of high blood pressure), diseases of the parents (diabetes mellitus, hypertension, CVD and cancer).

For some of the above-mentioned potential risk factors, missing values occurred in both the case and in the control group. Therefore the multiple imputation method was used [28]. Multiple imputation improves parameter estimates, standard errors, and test statistics when reconciling missing data. For information on BMI 9% of the data were missing. For the variables "blood pressure", "cholesterol levels" and the illnesses of parents, the answer "I do not know" was given by 5, 26 and 37 (father) or 20% (mother) of respondents, respectively. These variables were also processed using multiple imputation.

For the determination of a final multivariate model, backward selection was conducted. To compare the prevalence of CVD risk factors in the Aussiedler with that of the German population, we used data from other large studies and surveys [29-36].

## Results

For 103 of the 440 relatives of the potential cases, a contact telephone number was available. Among those, 58 interviews were performed (response rate including dropouts excluding cancelled due to illness, death or wrong contact data 73%). The remaining cases were contacted by mail only, through available addresses of relatives. Of those, 40 interviews were performed. The total number of cases for analysis is therefore 58 + 40 = 98. Calculation of an overall response rate is difficult since not all relatives of the cases may have received the letter or had a physical condition that allowed a response. Only few answered the letter, this indicates that they did not want to participate. Assuming half of all without telephone could have answered, we obtain an overall response estimate of (58 + 40)/(103 + 337/2) = 36.1%.

From the cohort, 680 potential controls were selected. Likewise, response rate of controls with telephone numbers was 77%, and the overall response rate under the same assumptions of potential response as for cases was 41%. The total number of interviewed controls for analysis is 250.

### Descriptive analysis

Descriptive analysis of the study population is given in Table 1. The participants of the study (N = 348) are predominantly male (N_case_male _= 70 (71.4%), N_case_female _= 28 (28.6%), N_control_male _= 183 (73.2%), N_control_female _= 67 (27.0%), Table 2). Median year of birth of cases and controls was 1935. Median age of death for cases is 65 years with a range from 31 to 88 years. At the time of the interview, the average age of the control group was 75 years (range 44-97 years). Education in cases and controls both in women and men is approximately equal. The majority of participants (N_case_male _= 49%, N_case_female _= 39%, N_control_male _= 52%, N_control_female _= 39%) were blue-collar workers or farmers, and the difference between cases and controls and women and men is relatively low. Housewives are 18% of the female case group and 10% of the female control group.

**Table 1:** Descriptive analysis of the study population

		cases	controls
		**N = 98**	**N = 250**

**Gender**	f	28 (28.6%)	67 (27.0%)

	m	70 (71.4%)	183 (73.2%)

**Year of birth**	1910-1919	7 (7.1%)	21 (8.4%)

	1920-1929	31 (32.0%)	71 (28.4%)

	1930-1939	33 (34.0%)	89 (36.0%)

	1940-1949	12 (12.2%)	29 (12.0%)

	1950-1959	11 (11.2%)	29 (12.0%)

	1960-1969	4 (4.1%)	11 (4.4%)

**Year of migration**	1990-1995	83 (84.7%)	209 (83.6%)

	1996-1999	10 (10.2%)	24 (9.6%)

	2000-2001	5 (5.1%)	17 (6.8%)

**Year of death**	1992-1995	20 (20.4%)	

	1996-1999	17 (17.3%)	

	2000-2003	35 (35.7%)	

	2004-2006	26 (26.5%)	

**Cause of death**	I20-I25 Ischaemic heart diseases	49 (48.9%)	

	I30-I52 Other forms of heart disease	19 (19.4%)	

	I60-I69 Cerebrovascular diseases	19 (19.4%)	

	other	12 (12.2%)	

	I00-I99 total	98 (100%)	

**Table 2:** Distribution by sex, univariate and adjusted odds ratios and 95% confidence intervals for selected variables among ethnic Germans

Variable	Males		Females		univariate^b^		multivariate^c^	
	
	Cases N (%^a^)	Controls N (%^a^)	Cases N (%^a^)	Controls N (%^a^)	OR	95%CI	OR	95%CI
**BMI (> 27.0 kg/m^2^)**	29 (41)	80 (44)	13 (46)	28 (42)	1.74	(1.05 - 2.90)		

**Alcohol consumption**	22 (31)	14 (8)	1 (4)	0 (0)	5.61	(2.65-11.87)	3.68	(1.58-8.58)

**Smoking (current or former)**	57 (81)	108 (59)	3 (11)	2 (3)	3.22	(1.60 - 6.12)	3.07	(1.42-6.62)

**physical activity (intensive)**	3 (4)	16 (9)	0 (0)	6 (9)	0.29	(0.08 - 1.01)		

**Daily Fruit and vegetable consumption**	38 (54)	135 (74)	24 (86)	49 (73)	0.64	(0.39 - 1.04)		

**Sweets (little)**	25 (36)	103 (56)	9 (32)	29 (43)	0.46	(0.27 - 0.76)	0.34	(0.18-0.64)

**Salt (little)**	37 (53)	124 (68)	21 (75)	52 (78)	0.50	(0.37 - 0.97)		

**Work (physically demanding)**	56 (80)	137 (75)	18 (64)	46 (69)	1.13	(0.65 - 1.95)		

**Low education**	34 (49)	76 (42)	16 (57)	35 (52)	1.06	(0.46 - 2.43)		

**Diabetes mellitus (yes)**	16 (23)	17 (9)	5 (18)	8 (12)	2.53	(1.31 - 4.88)	3.29	(1.46-7.39)

**High blood pressure (yes)**	42 (64)	100 (57)	18 (69)	43 (66)	1.26	(0.75 - 2.12)		

**High cholesterol level (yes)**	23 (41)	19 (13)	3 (20)	11 (23)	3.10	(1.58 - 6.07)	2.32	(1.11-4.88)

**Drug treatment of hypertension (yes)**	36 (51)	89 (49)	18 (64)	39 (58)	1.17	(0.72 - 1.80)		

**Diabetes mellitus of the parents (yes)**	14 (28)	7 (4)	1 (5)	3 (5)	4.53	(1.75 - 11.74)		

**High blood pressure of the parents (yes)**	18 (37)	39 (23)	5 (28)	20 (32)	1.71	(0.75 - 3.80)		

**CVD of the parents (yes)**	15 (33)	47 (28)	4 (24)	23 (37)	1.35	(0.60 - 2.63)		

**Cancer of the parents (yes)**	11 (23)	34 (20)	0(0)	17 (27)	0.93	(0.46 - 1.89)		

^a ^percentage exposed of all non-missings

^b ^based on conditional logistic regression, stratified by sex, year of birth, year of migration

^c ^based on conditional logistic regression, variables selected by backward selection method

The distribution of relevant variables is given in Table 2. Both in women and in men, there are more ever smokers in the cases than in controls. Whereas about 80% of men in the case group have ever smoked, only about 60% of men ever smoked in the control group. The overall difference between cases and controls is significant in both sexes (p_m _< 0.0001, p_w _= 0.01). Women, both in the case group and in the control group, smoked significantly less than men.

Overall, the type of alcohol consumed differs significantly between cases and controls (p < 0.0001). Regarding overall consumption of alcohol, women in both cases and controls drank less alcohol than men. The alcohol consumption of women in the control group was only slightly lower than that of women from the case group. However, male cases drank significantly more alcohol than women and male controls. Male controls who drink alcohol consumed on average 0.3 liters of pure alcohol per month, while cases consumed more than 0.7 liters. Calculation was based on questions about the consumption of vodka, beer and wine assuming 37.5 vol %, 5 vol % and 12 vol % of alcohol, respectively. The differences between cases and controls regarding alcohol consumption at special occasions are relatively low (p = 0.85, p_m _= 0.58, p_w _= 0.05), so that the major differences in total alcohol consumption are due to a constantly higher level of alcohol consumption of the cases.

There are clear differences in diet between cases and controls. Cases consumed significantly more meat and meat products than controls (95% vs. 80% daily consumption, p = 0.004). There are also clear differences in the consumption of chocolate and confectionery. Here, cases showed a significantly greater consumption than the controls, which also applies to salty snacks. In the case group white bread was preferred to whole grain bread while it was the opposite in the control group (64% vs. 34% white bread and 20% vs. 42% whole grain bread).

BMI for controls and cases, both in women and in men, was relatively uniform, with means of 27 (controls male), 28 (control women) and 28 (cases male), and 29 (cases women) kg/m^2^. Approximately 90% of the cases were not at all active in sports, while this applies to about 70% of the controls. More than 8% of the controls practiced sport intensively. For cases, this was true only for 4% of the men, while none of the women answered affirmatively. The difference in intensity of the physical activities between cases and controls is also significant (p = 0.006). When asked about their profession, most of the cases and the controls claimed that their work was physically demanding.

Cases had more often severe diseases than the controls. In all categories concerning the questions on health, the incidence of disease is higher in cases than in controls. The differences are significant for the following diseases: stroke (p < 0.0001), diabetes mellitus (p = 0.01), cancer (p = 0.01), circulatory disturbances of the heart (p < 0.0001), circulatory disturbances in the legs (p = 0.002), heart disease (p < 0.0001) and cholesterol values (p = 0.002).

### Analytical evaluation

Table 2 shows univariate odds ratios (OR) for all considered potential risk factors (see above) with 95% confidence intervals (CI). Significant factors with particularly high univariate odds ratios are alcohol consumption with OR = 5.61 [95% CI, 2.65-11.87], smoking with OR = 3.22 [95% CI, 1.60-6.12], diabetes mellitus with OR = 2.53 [95% CI; 1.31-4.88], cholesterol with OR = 3.10 [95% CI, 1.58-6.07], and diabetes of the parents with OR = 4.53 [95% CI, 1.75-11.74].

After performing a backward-selection, the results given in Table 2 are obtained. This best model includes five risk factors, four of them mentioned above discussing the univariate ORs (alcohol, smoking, diabetes, cholesterol). Additionally included is the consumption of sweets, while parental diabetes is no longer a significant risk factor. For alcohol consumption and smoking, OR = 3.68 [95% CI, 1.58-8.58] and OR = 3.07 [95% CI, 1.42-6.62], respectively. For diabetes mellitus and high cholesterol values, OR = 3.29 [95% CI, 1.46-7.39] and OR = 2.32 [95% CI, 1.11-4.88], respectively. The almost complete abdication of sweets is associated with a protective effect, OR = 0.34 [95% CI, 0.18-0.64].

For men alone (data not shown in Table), a slightly different result is obtained (multivariate analysis). Cholesterol is no longer an important risk factor, while parental diabetes mellitus becomes important, with OR = 5.65 [95% CI; 1.62-19.70]. For alcohol consumption, OR = 4.26 [95% CI; 1.65-11.00]. OR = 3.16 [95% CI; 1.31-7.63] for smoking, OR = 4.12 [95% CI; 1.43-11.87] for diabetes mellitus and OR = 0.32 [95% CI; 0.14-0.74] for the consumption of sweets, respectively. For women alone such a model cannot be estimated as no woman of the controls consumes alcohol.

To compare the prevalence of the risk factors in the Aussiedler with the prevalence of these risk factors in the native German population, data from the literature were used [29-36] (Table 3). Alcohol consumption of the cases was calculated in g/day and compared to the percentage of men and women found in the literature [32] drinking more than 20 and 10 g/day, respectively. Age-weighted averages (according to the age distribution of the controls) of the literature data were calculated. Concerning alcohol consumption, both male and female Aussiedler reported less drinking than the native German population whereas smoking behavior - at least for men who constitute the majority in this study - is comparable. Fruit and vegetable consumption show no significant differences. More Aussiedler reported no physical activity at all. Hypertension, high cholesterol and diabetes mellitus also have lower prevalences in Aussiedler. Risk factors with a higher prevalence in Aussiedler are overweight in females and physical inactivity.

**Table 3:** Prevalence of potential risk factors among controls and comparison with the native German population

Potential risk factors	Prevalence of potential risk factors among Aussiedler (controls) in %		Prevalence of the native German population in %		Literature/comments
		
	Male	Female	Male	Female	
Alcohol (> 20 g/day for men) Alcohol (> 10 g/day for women)	**11.9^a^**	**0**	**30.5**	**13.3**	[32]^c^

Smoking (yes)	**59.0**	**3.0**	**58.9**	**24.4**	[35]^c^

Diabetes mellitus (yes)	**9.3^b^**	**10.8^b^**	**17.9/9.8**	**19.3/13.8**	[30]^c^,
					
					new/old federal states

Cholesterol (yes)	**9.3^b^**	**18.9^b^**	**13.1**	**16.2**	[31]^c^
					
					> 300 mg/100 ml,
	
			**13.1**	**14.3**	[35]

BMI (> 25.0 kg/m^2^)	**68.4**	**76.1**	**69.7**	**56.0**	[29]^c^
					
					> 25 kg/m2

High blood pressure (yes)	**57.8**	**66.2**	**48.4**	**50.9**	[34]^c^
					
					12 months prevalence, yes
	
			**79.4**	**75.9**	[33]
					
					SBP ≥ 140 mmHg and/or
					
					DBP ≥ 90 mmHg

No regular physical activity	**71.6**	**56.7**	**49.6**	**46.0**	[34]^c^

Fruit and vegetables (daily)	**73.8**	**73.1**	**67.5**	**81.9**	[34]^c^

^a ^Only taking into account the age group 45-79 for comparison with literature

^b ^Only taking into account the age group 40-79 for comparison with literature

^c ^Age-weighted averages, according to age distribution in control group

## Discussion

There are few data on the health behavior of "Aussiedler", second largest migrant group in Germany. The analysis presented here represents a first attempt to identify the risk factors of fatal cardiovascular diseases, to quantify their effects among Aussiedler, and to compare the risk factor prevalences with that of the native German population, in order to provide an explanation why earlier studies have shown a lower cardiovascular disease mortality in Aussiedler compared to native Germans.

Major risk factors for cardiovascular diseases are known (see, for example, [37]). In this study, these risk factors were confirmed to be also relevant in the group of Aussiedler. The results on risk factors and the magnitude of their effects are largely consistent with earlier studies on risk factors for this disease. The occurrence of stroke, diabetes mellitus, cancer, circulatory disturbances, and circulatory problems in the legs, heart disease and high cholesterol levels was significantly higher within cases in comparison to controls. The difference with respect to smoking habits and total alcohol consumption between cases and controls is also significant. Differences were also evident in the diet between cases and controls e.g. cases consumed significantly more meat and meat products.

Comparing the univariate with the adjusted ORs (Table 2), confounding of major risk factors was observed, resulting in reduced adjusted ORs, for example in the consumption of alcohol (OR = 5.61 vs. OR = 3.52). The effect of several other factors, for example physical activity, was no longer significant. The observed finding of a reduced risk with low consumption of sweets must not be overinterpreted. It could be a chance finding, or it is also possible that low sweet consumption is associated with several factors which all contribute to CVD risk, such as consumption of more healthy foods, physical activity, and also of BMI. Regarding alcohol, a moderate consumption has consistently been shown to be protective, and only high consumption is associated with an increased risk. Our sample is too small to perform detailed dose-response analyses, and since we can assume some underreporting of the true alcohol consumption, we think that the cutpoint chosen is appropriate for categorising doses with a high risk.

The comparision of major risk factors to that of the native German population showed a lower prevalence for alcohol consumption. We expected a higher prevalence because of the high alcohol consumption in the former Soviet Union, especially binge drinking. It appears that this is not the case. Strobl and Kühnel also found generally low alcohol consumption among Aussiedler with the exception of adolescents [38]. Smoking appears similarly distributed in men, and with a lower prevalence in females. High cholesterol also has lower prevalences in Aussiedler. For hypertension, different results were found in the literature, so that a comparison is difficult. In contrast, overweight in females and low physical inactivity are more among Aussiedler.

However, due to the small sample size, differences to the German reference data (Table 3) are rarely significant. Overall, these results are in line with an observed lower CVD mortality in Aussiedler compared to native Germans.

However, results are in contrast to studies from the USA, Canada and Sweden, which show that the mortality of migrants is determined by the mortality in their country of origin [39-41]. For Jews in Moscow, on the other hand, a lower mortality rate than the Moscow-average was determined and has been associated with certain ways of living [42]. Although the socio-economic status, measured by conventional criteria, such as income, is lower for Aussiedler than for native Germans, there are signs that resettlers have a high satisfaction with their life [43]. Selection with respect to the health of Aussiedler alone is unlikely to explain the observed effect of a lower mortality seen in the previous paper. However, it should be noted that there could be a selection by healthiness especially among older immigrants, while in the younger group, in which diseases are still much less obvious, this selection might be missing. It is known that subgroups may have a significantly different life expectancy than the average population, as is shown by example in different districts of Chicago where life expectancy differs up to 20 years [44]. Whether such a big difference between Aussiedler living in Russia and local Russians exist is unknown [45].

This study has a number of limitations. As a nested case-control study, it was not possible to increase the sample size and the number of cases is limited. The total number of cases is about 10% of the total members of the cohort with a CVD death. Therefore, the power to detect rare factors or factors with a moderate risk is rather low. The response rate is high among those who could be contacted by telephone, but overall relatively low. However, the rate may be underestimated because the denominator could not be given exactly. The comparison of risk factor prevalences between the Aussiedler and Germans has some limitations in the data for comparison. Some recall bias is likely, however, it is not possible to quantify this bias. Unfortunately, we don't have any data about a possible difference in healthiness or socioeconomic status between participants with or without living relatives. However, most of the controls (63%) do also have living relatives as can be extracted from the cohort data. We can also assume that several of those without a relative in our database in fact have one or more relatives, which were unknown to us. Therefore, we assume that a bias due to this aspect is small. We are aware that a possible reporting bias is one of the strongest limitations of our study. However, as is shown in the study of Nelson et al. [46], who used a dual interview protocol in a case-control study where control subjects and their proxy respondents were interviewed, the reliability of proxy-derived data was excellent for demographic and body habitus measures and all aspects of cigarette smoking history. Proxy reliability was only somewhat lower for questions regarding medications and hormone preparations, alcolhol consumption, and recreational physical activity. The causes of death for cases are a broad heterogeneous group of conditions with possibly different sets of risk factors. Alcohol consumption is reported to be only a rather protective factor for Myocardial infarction and cerebrovascular diseases in low doses, and a high risk factor for high doses [47]. The very high Odds Ratio for alcohol consumption (> 20 g/day in males and > 10 g/day in females) found in our study is therefore a little surprising and should be further evaluated. For this particular factor, a reporting bias must be considered. The causes of death are not based on review of medical records or adjudicated causes of death but solely on death reports and coding with its possible inaccuracies. For comparison of risk factor prevalences with the German population we used results from several earlier surveys. Although these data were not obtained in an identical way compared to this study, we think a comparison is appropriate. We have selected those surveys which we consider as most appropriate.

## Conclusions

Results of this study may partly explain the observation of earlier studies that CVD mortality is significantly lower in Aussiedler than in Germans. The reported lower prevalences of known risk factors of CVD such as alcohol consumption, high cholesterol, diabetes and smoking (in women) could contribute to a lower risk of CVD. Although the relatively low alcohol consumption is surprising, given the known high alcohol consumption in the former Soviet Union, it is possible that the Aussiedler had behavioral patterns that differed from the native population in the former Soviet Union. A new prospective cohort study on this group of migrants has just started and will elucidate this issue.

## Abbreviations

KORA study: Cooperative health research in the Augsburg region; 95%-CI: 95 percent confidence interval; SMR: Standardized mortality ratio; CVD: Cardiovascular and cerebrovascular diseases; NRW: North Rhine-Westphalia; ICD: International statistical classification of diseases and related health problems; SAS: Statistical analysis system; OR: Odds ratio; BMI: Body mass index; SBP: systolic blood pressure; DBP: Diastolic blood pressure

## Competing interests

The authors declare that they have no competing interests.

## Authors' contributions

EK collected the data, performed the calculations, developed the figures and drafted the manuscript. VW provided knowledge regarding statistical and methodological problems. HB conceived the study. All authors read and approved the final manuscript.

## Pre-publication history

The pre-publication history for this paper can be accessed here:

http://www.biomedcentral.com/1471-2458/12/190/prepub

## References

[B1] Bundesministerium des Innern (BMI) Migration und Integration. Aufenthaltsrecht, Migrations- und Integrationspolitik in Deutschland Aktuelle Eckdaten 2008 26

[B2] Zeeb H Razum O Epidemiologische Studien in der Migrationsforschung Bundesgesundheitsblatt Gesundheitsforschung Gesundheitsschutz 2006 49 845 852 10.1007/s00103-006-0017-5 16937322

[B3] Pfetsch B In Russia we were Germans, and now we are Russians. - Dilemmas of identity formation and communication among German-Russian Aussiedler FS III 99-103 Veröffentlichungsreihe der Abteilung Öffentlichkeit und soziale Bewegungen des Forschungsschwerpunktes Sozialer Wandel, Institutionen und Vermittlungsprozesse des Wissenschaftszentrums Berlin für Sozialforschung 1999 http://skylla.wzb.eu/pdf/1999/iii99-103.pdf

[B4] Aparicio ML Döring A Mielck A Holle R und die KORA Studiengruppe Unterschiede zwischen Aussiedlern und der übrigen deutschen Bevölkerung bezüglich Gesundheit, Gesundheitsversorgung und Gesundheitsverhalten: eine vergleichende Analyse anhand des KORA-Surveys 2000 Soz Praventivmed 2005 50 107 118 10.1007/s00038-004-3088-9 15900963

[B5] Becher H Razum O Kyobutungi C Laki J Ott JJ Ronellenfitsch U Winkler V Mortalität von Aussiedlern aus der ehemaligen Sowjetunion. Ergebnisse einer Kohortenstudie Dtsch Arztebl 2007 104 23 A-1655 A-1661

[B6] Razum O Zeeb H Akgun HS Yilmaz S Low overall mortality of Turkish residents in Germany persists and extends into a seond generation: merely a healthy migrant effect? Trop Med Int Health 1998 3 297 303 10.1046/j.1365-3156.1998.00233.x 9623931

[B7] Razum O Geiger I Zeeb H Ronellenfitsch U Gesundheitsversorgung von Migranten Dtsch Arztebl 2004 101 43 A-2882 A-2887

[B8] Junghanss T Asylsuchende und Flüchtlinge: Gesundheitsversorgung einer komplexen Minderheit Soz Praventivmed 1998 43 11 17 10.1007/BF01299236 9544466

[B9] Tselmin S Korneblum W Reimann M Bormstein SR Schwarz PEH The Health Status of Russian-speaking Immigrants in Germany Horm Metab Res 2007 39 858 861 10.1055/s-2007-993153 18075968

[B10] Bobak M Marmot M East-West mortality divide and its potential explanations: proposed research agenda BMJ 1996 312 421 425 10.1136/bmj.312.7028.421 8601115 PMC2350098

[B11] Ciment J Life expectancy of Russian men falls to 58 BMJ 1999 319 468 10454391 10.1136/bmj.319.7208.468a PMC1116380

[B12] Ginter E Cardiovascular risk factors in the former communist countries Analysis of 40 European MONICA populations Eur J Epidemiol 1995 11 199 205 10.1007/BF01719488 7672076

[B13] Lancet T Health in Russia is broke, but who is to fix it? Lancet 1999 353 30 337 9950431 10.1016/s0140-6736(99)90019-3

[B14] Leon DA Chenet L Shkolnikov VM Zakharov S Shapiro J Rakhmanova G Vassin S McKee M Huge variation in Russian mortality rates 1984-94: artefact, alcohol, or what? Lancet 1997 350 383 388 10.1016/S0140-6736(97)03360-6 9259651

[B15] Federal state statistics service. Death rates by main classes of causes of death (deaths per 100 000 population) http://www.gks.ru/bgd/regl/b08_12/IssWWW.exe/stg/d01/05-07.htm

[B16] Adler NE Ostrove JM Socioeconomic status and health: what we know and what we don't Ann N Y Acad Sci 1999 896 3 15 10.1111/j.1749-6632.1999.tb08101.x 10681884

[B17] Tyroler HA The influence of socioeconomic factors on cardiovascular disease risk factor development Prev Med 1999 29 36 40 10.1006/pmed.1998.0441 10641816

[B18] Marmot MG Socio-economic Factors in Cardiovascular Disease J Hypertens Suppl 1996 14 204 205 9120680

[B19] Ronellenfitsch U Razum O Deteriorating health satisfaction among immigrants from Eastern Europe to Germany Int J Equity Health 2004 3 4 doi:10.1186/1475-9276-3-4 10.1186/1475-9276-3-4 PMC441401 15193155

[B20] Ronellenfitsch U Cardiovascular Mortality among Ethnic German Immigrants from the Former Soviet Union to Germany: a retrospective cohort study Inauguraldissertation der Medizinischen Fakultät Heidelberg der Ruprecht-KarlsUniversität 2005

[B21] Kawabata H Tran M Hines P Using SAS ^® ^to Match Cases for Case Control Studies SUGI 29 2004 Princeton, New Jersey 173 29

[B22] Parsons LS Ovation Research Group Performing a 1:N Case-Control Match on Propensity Score SUGI 29 2004 Seattle, Washington 165 29

[B23] Parsons LS Using SAS^® ^Software to Perform a Case-Control Match on Propensity Score in an Observational Study SUGI 25 2000 Cardiovascular Outcomes Research Center, Seattle, WA 225 25

[B24] Rosenbaum PR Rubin D The central role of the propensity score in observational studies for causal effects Biometrilca 1983 70 1 41 55 10.1093/biomet/70.1.41

[B25] Holle R Happich M Löwel H Wichmann HE MONICA/KORA Study Group KORA - A Research Platform for Population Based Health Research KORA - Eine Forschungsplattform für bevölkerungsbezogene Gesundheitsforschung Gesundheitswesen 2005 67 1 19 S25 10.1055/s-2005-858235 16032513

[B26] Kleinbaum GD Klein M Logistic Regression A Self-Learning Text Introduction to Logistic Regressio, Volume Kapitel 1 2002 2 Berlin Heidelberg: Springer-Verlag New York 13 50

[B27] Robert Koch-Institut (RKI) Bundes-Gesundheitssurvey: Alkohol Konsumverhalten in Deutschland Beiträge zur Gesundheitsberichterstattung des Bundes 2003 6

[B28] Spratt M Carpenter J Sterne JAC Carlin JB Heron J Henderson J Tilling K Strategies for Multiple Imputation in Longitudinal Studies Am J Epidemiol 2010 172 478 487 10.1093/aje/kwq137 20616200

[B29] Statistisches Bundesamt Mikrozensus - Fragen zur Gesundheit - Körpermaße der Bevölkerung 2009 Wiesbaden 2011 15

[B30] Robert Koch-Institut (RKI) Diabetes mellitus. Gesundheitsberichterstattung des Bundes Heft 24. Berlin 2005 11

[B31] Robert Koch-Institut (RKI) Gesundheitsberichterstattung des Bundes. Gesundheit in Deutschland Berlin 2006 116

[B32] Robert Koch-Institut (RKI) Alkoholkonsum und alkoholbezogene Störungen Heft 40. Berlin 2008 12

[B33] Robert Koch-Institut (RKI) Hypertonie Heft 43. Berlin 2008 11

[B34] Robert Koch-Institut (RKI) Beiträge zur Gesundheitsberichterstattung des Bundes Daten und Fakten: Ergebnisse der Studie „Gesundheit in Deutschland aktuell 2009" Berlin 2011 106 109 112 128

[B35] DKFZ - Deutsches Krebsforschungszentrum Tabakatlas Deutschland 2009 Heidelberg 2009 28

[B36] Thefeld W Verbreitung der Herz-Kreislauf-Risikofaktoren Hypercholesterinämie, Übergewicht, Hypertonie und Rauchen in der Bevölkerung. Robert Koch-Institut, Berlin Bundesgesundheitsblatt Gesundheitsforschung Gesundheitsschutz 2000 43 415 423 10.1007/s001030070047

[B37] Grundy SM Pasternak R Greenland P Smith S Fuster V Assessment of Cardiovascular Risk by Use of Multiple-Risk-Factor Assessment Equations. A Statement for Healthcare Professionals from the American Heart Association and the American College of Cardiology Circulation 1999 100 1481 1492 http://www.circulationaha.org 10500053 10.1161/01.cir.100.13.1481

[B38] Strobl R Kühnel W Dazugehörig und ausgegrenzt. Analysen zu Integrationschancen junger Aussiedler Weinheim/München 200022359355

[B39] Hammar N Kaprio J Hagstrom U Alfredsson L Koskenvuo M Hammar T Migration and mortality: a 20 year follow up of Finnish twin pairs with migrant co-twins in Sweden J Epidemiol Community Health 2002 56 362 366 10.1136/jech.56.5.362 11964433 PMC1732140

[B40] Nair C Nargundkar M Johansen H Strachan J Canadian cardiovascular disease mortality: first generation immigrants versus Canadian born Health Rep 1990 2 203 228 2101285

[B41] Singh GK Siahpush M Ethnic-immigrant differentials in health behaviors, morbidity, and cause-specific mortality in the United States: an analysis of two national data bases Hum Biol 2002 74 83 109 10.1353/hub.2002.0011 11931581

[B42] Shkolnikov VM Andreev EM Anson J Mesle F The peculiar pattern of mortality of Jews in Moscow, 1993-95 Popul Stud (Camb) 2004 58 311 329 10.1080/0032472042000272366 15513286

[B43] Weick S Zuwanderer in Deutschland optimistisch. Untersuchung zu Lebensbedingungen, Integration und Zufriedenheit bei Migranten ZUMA Publikation 1996 16 ISI: Informationsdienst soziale Indikatoren Sozialberichterstattung, gesellschaftliche Trends, aktuelle Informationen 1 4

[B44] Wilson M Daly M Life expectancy, economic inequality, homicide, and reproductive timing in Chicago neighbourhoods BMJ 1997 314 1271 1274 10.1136/bmj.314.7089.1271 9154035 PMC2126620

[B45] Ronellenfitsch U Kyobutungi C Becher H Razum O All-cause and Cardiovascular mortality among ethnic German immigrants from the Former Soviet Union: a cohort study BMC Public Health 2006 6 16 10.1186/1471-2458-6-16 16438727 PMC1403762

[B46] Nelson LM Longstreth WT Koepsell TD Checkoway H van Belle G Completeness and Accuracy of Interview Data from Proxy Respondents: Demographic, Medical, and Life-style Factors Epidemiology 1994 5 2 204 217 10.1097/00001648-199403000-00011 8172996

[B47] Ronksley PE Brien SE Turner BJ Mukamal KJ Ghali WA Association of alcohol consumption with selected cardiovascular disease outcomes: a systematic review and meta-analysis BMJ 2011 342 d671 10.1136/bmj.d671 21343207 PMC3043109

